# Efficacy of endoscopic retrograde appendicitis therapy in children with suppurative vs. simple appendicitis: a retrospective cohort study

**DOI:** 10.3389/fped.2026.1729439

**Published:** 2026-03-25

**Authors:** Ning Xue, Xu-Xia Wei, Jun-Jie Xu, Lu Yang, Yun-Ping Tang

**Affiliations:** 1 Department of Gastroenterology, Children’s Hospital Affiliated to Shandong University (Jinan Children’s Hospital), Jinan, Shandong, China; 2 Shandong Provincial Clinical Research Center for Children’s Health and Disease, Jinan, Shandong, China

**Keywords:** appendiceal perforation, children, endoscopic retrograde appendicitis therapy (ERAT), retrospective cohort study, suppurative appendicitis, therapeutic efficacy

## Abstract

**Objective:**

To evaluate the efficacy of endoscopic retrograde appendicitis therapy (ERAT) in the treatment of pediatric suppurative appendicitis.

**Methods:**

In this retrospective cohort study, children undergoing ERAT for acute appendicitis between January 2019 to March 2025 were categorized according to disease severity into suppurative or simple appendicitis groups. Data encompassing baseline characteristics, preoperative profiles, procedural outcomes, and follow-up were collected and subjected to intergroup comparison.

**Results:**

The study included 61 children (31 suppurative, 30 simple). Two patients in the suppurative group were excluded from the follow-up analysis due to missing data at the one-week time point. Preoperative fever, vomiting, appendicoliths, and inflammatory markers were significantly more common in the suppurative group (all *P* < 0.05). The mean operative time was significantly longer in the suppurative group (105.5 ± 34.1 vs. 86.3 ± 34.4 min, *P* < 0.001). While ERAT achieved a 100% success rate in simple appendicitis, the success rate was significantly lower in the suppurative group (74.2% vs. 100%, *P* < 0.001), with appendiceal perforation being the primary complication (12.9%, 4/31). Treatment failure led to appendectomy in 6 patients (20.7%) with suppurative appendicitis. Among initially successful cases, the suppurative group had a significantly prolonged recovery, including longer time to pain relief and hospital stay (both *P* < 0.001). At 6-month follow-up, the recurrence rate was numerically higher in the suppurative group (21.7% vs. 10.0%), although the difference was not statistically significant (*P* = 0.117). Parental satisfaction scores trended lower and pain scores higher in the suppurative group during follow-up.

**Conclusion:**

For suppurative appendicitis, particularly with a thin-walled appendix or large fecalith, ERAT is not recommended due to low success rates and perforation risk; laparoscopic appendectomy remains the standard. ERAT is a safe, effective organ-preserving option only for carefully selected patients with simple appendicitis or select non-necrotizing fecalith impaction.

## 1 Introduction

Acute appendicitis is a common abdominal emergency in children. The understanding of appendicitis dates back to the 16th century, but it was not until the late 19th century that systematic treatment emerged. In 1883, Abraham Groves provided the first accurate description of the clinical manifestations and pathological changes of appendicitis and introduced the concept of appendectomy, thereby laying the foundation for its modern management (1). By the early 20th century, open appendectomy (OA) had become the standard treatment, offering direct visualization and procedural simplicity, albeit with drawbacks such as significant tissue trauma and prolonged recovery (2). The advent of minimally invasive surgery in the 1980s led to the development of laparoscopic appendectomy (LA). Semm performed the first LA in 1983, marking the beginning of a new era in minimally invasive appendicitis treatment (3). Extensive studies have demonstrated that LA offers several advantages over OA, including reduced surgical trauma, less postoperative pain, faster recovery, and fewer complications, making it especially suitable for pediatric patients (4–6). As a result, LA is now the preferred surgical approach for appendicitis (7). Although appendectomy has significantly reduced mortality rates, the rate of negative appendectomies remains considerable, reported at 8% in adults and 3.3% in children (8).

In recent years, the role of the appendix in the development of intestinal immunity and microbial defense has gained increasing recognition. Studies have revealed significant correlations between appendectomy and disruptions in gut microbiota homeostasis, gastrointestinal inflammation, and even tumorigenesis (9–11). Although appendectomy remains the standard of care for pediatric appendicitis, there is growing interest in appendix-preserving treatment strategies. In 2012, Liu et al. (12) introduced a novel minimally invasive endoscopic approach known as endoscopic retrograde appendicitis therapy (ERAT), which integrates endoscopic retrograde appendiceal radiography, irrigation, and stenting. After more than a decade of refinement, ERAT has gained acceptance in several East Asian countries and is increasingly being applied in the diagnosis and management of pediatric appendicitis (13–15). The emergence of ERAT marks the arrival of an “ultra-minimally invasive” era in appendicitis treatment, offering a new clinical alternative and enabling elective surgical planning and other interventions. Current research on ERAT has predominantly focused on simple appendicitis, with limited reports concerning its use in suppurative appendicitis. We evaluated the outcomes of ERAT in pediatric suppurative appendicitis cases at our center. The findings aim to provide evidence-based guidance for determining the optimal treatment window and technical approach.

## 2 Materials and methods

### 2.1 Study design and patients

This retrospective cohort study included pediatric patients diagnosed with acute appendicitis who underwent endoscopic retrograde appendicitis therapy (ERAT) at the Department of Gastroenterology, Children's Hospital Affiliated to Shandong University, between January 2019 and March 2025. Patients were categorized into two groups: the study group consisted of 31 children with a clinical diagnosis of suppurative appendicitis, while the control group comprised 30 children diagnosed with simple appendicitis during the same period. The study protocol received approval from the Institutional Ethics Committee of Jinan Children's Hospital (Approval No. SDFE-IRB/T-2025126). Written informed consent was obtained from the parents or legal guardians of all participants.

### 2.2 Inclusion and exclusion criteria

The inclusion criteria were as follows: (1) age between 3 and 16 years; and (2) a clinical diagnosis of acute appendicitis corroborated by medical history, physical examination, laboratory tests, and imaging findings (ultrasound or computed tomography).

Exclusion criteria included: (1) evidence of complicated appendicitis, such as perforation, gangrene, intra-abdominal abscess, or diffuse peritonitis; (2) other identified causes of acute abdominal pain (e.g., pancreatitis, cholecystitis, intussusception, or abdominal Henoch–Schönlein purpura); (3) contraindications to general anesthesia or colonoscopy.

### 2.3 Instruments

All ERAT procedures were performed using a standard video colonoscope (Olympus PCF-260; outer diameter: 12.9 mm, working channel: 3.2 mm). The selection of this model was based on a comprehensive evaluation of the child's physique, preoperative imaging findings, and procedural requirements. For older pediatric patients typically weighing more than 15 kg and without severe colonic anomalies, we deemed that the use of this standard colonoscope could ensure procedural safety while fully leveraging the advantage of its large working channel to guarantee the effective completion of therapeutic steps such as stone extraction and drainage.

### 2.4 ERAT procedure details

All patients underwent preoperative assessment and standard bowel preparation after admission. A fasting period of at least 6 h was mandated prior to the procedure. ERAT was performed under intravenous general anesthesia using propofol. Perioperative antibiotics and supportive care were administered based on the patient's clinical status and laboratory parameters.

Under general anesthesia, all ERAT procedures were performed by an endoscopist with a minimum of ten years of experience in pediatric interventional endoscopy. Based on preoperative abdominal ultrasound findings and the procedural expertise accumulated over time, two technical approaches were employed. In the early stages of the ERAT program, the ultrasound-guided technique (UG-ERAT) was predominantly used. With growing experience and refinement of the technique, direct-vision ERAT (DV-ERAT) became the preferred approach for the majority of cases.

#### 2.4.1 UG-ERAT

Indications: Patients with ultrasonographically confirmed acute appendicitis exhibiting either: (1) intraluminal hyperechoic foci with posterior acoustic shadowing (appendicoliths), or (2) marked appendiceal distension (>10 mm diameter) containing turbid fluid.

Procedure: The procedure began with a cap-assisted colonoscopy to reach the cecum and localize the appendiceal orifice. Simultaneously, a dedicated sonographer performed real-time ultrasonography of the right lower quadrant using a high-frequency linear transducer. Under combined endoscopic-ultrasonic guidance, a guidewire was advanced into the appendiceal lumen; sonographic confirmation was obtained once the wire passed any obstructing appendicolith or reached the appendiceal tip. Subsequent steps included copious saline irrigation, endoscopic retrieval of appendicoliths using a basket or balloon catheter, and, if indicated for luminal stenosis, placement of a pancreaticobiliary plastic stent. Real-time ultrasound monitoring throughout the procedure confirmed successful clearance of intraluminal contents, appendiceal decompression, and optimal stent deployment.

**Figure 1:**
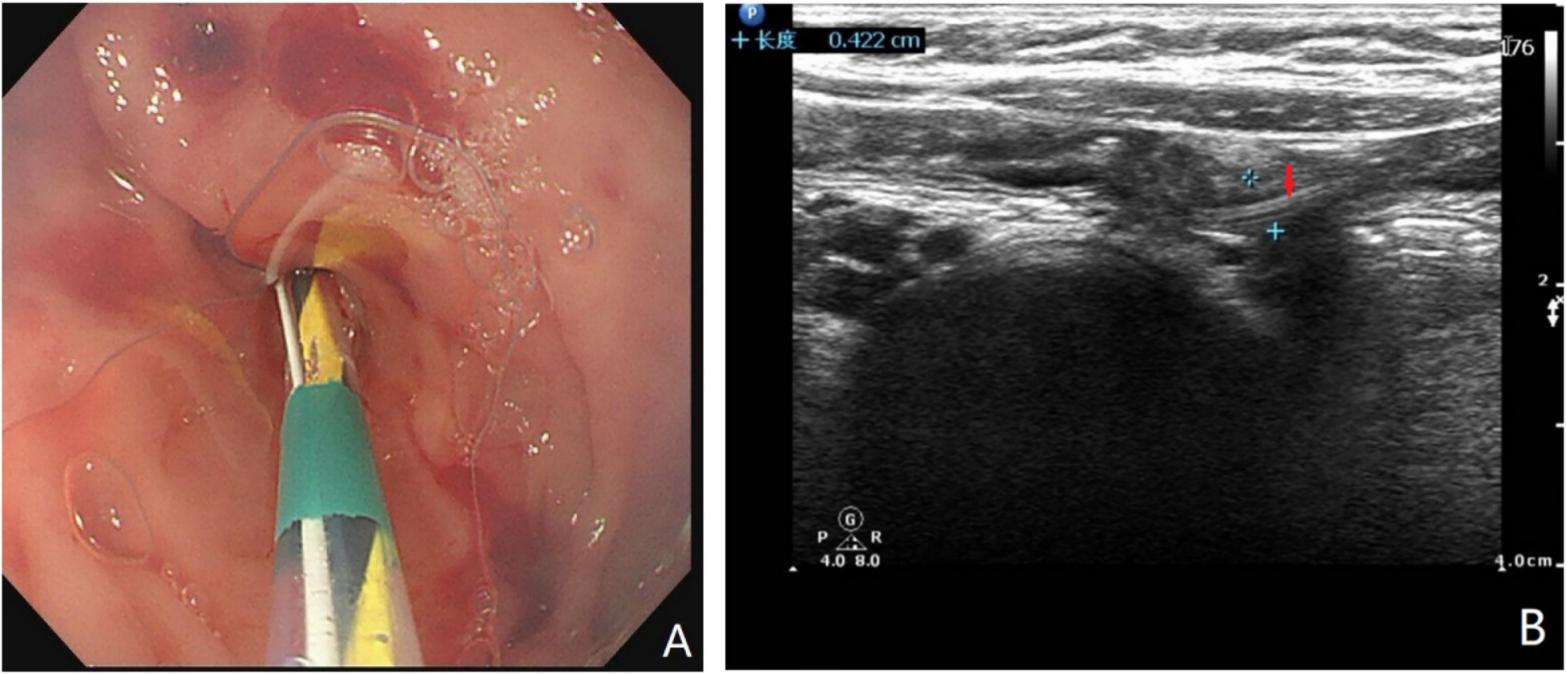
Intraoperative findings during UG-ERAT. **(A)** Endoscopic view showing a sphincterotome advanced over a guidewire into the appendiceal orifice. **(B)** Corresponding real-time ultrasonographic image confirming guidewire placement within the appendix (red arrow).

For UG-ERAT, a sphincterotome was advanced over the guidewire into the appendiceal lumen (Figures 1A,B). Saline irrigation was performed to clear fecal debris, with real-time ultrasonography used to confirm guidewire positioning in the distal appendix and assess irrigation adequacy.

#### 2.4.2 DV-ERAT

Indications: Acute appendicitis confirmed by preoperative ultrasound, without definitive appendicoliths or significant appendiceal distension (typically diameter <10 mm).

Procedure: Following the same preparatory steps as the UG-ERAT protocol, a colonoscope fitted with a transparent cap was advanced to the cecum. After identifying and optimally exposing the appendiceal orifice, a disposable digital colonoscope was introduced through the working channel and carefully advanced into the appendiceal lumen under continuous saline irrigation for visualization and dilation. This approach enabled real-time, intraluminal inspection of the mucosal surface, permitting direct assessment of inflammatory changes such as congestion, edema, or erosion, as well as the identification of any small or obscured appendicoliths. Upon reaching the appendiceal tip, thorough irrigation was performed until the effluent was clear. Any identified appendicoliths were removed using a combination of targeted irrigation, endoscopic retrieval basket, or balloon catheter extraction.

Completion Criteria: The procedure was concluded only after complete luminal clearance and the absence of residual debris or stones were confirmed under direct endoscopic vision, following which the colonoscope was safely withdrawn.

### 2.5 Technical selection principles

The selection between UG-ERAT and DV-ERAT was guided by a comprehensive, risk-adapted strategy that evolved with procedural experience. As a foundational safety measure, all patients were evaluated by the pediatric surgery department prior to the procedure. In the early phase, the primary determinant for selecting UG-ERAT was the presence of high-risk features identified on preoperative ultrasound, most notably impacted appendicoliths, where real-time sonographic guidance was deemed crucial for enhancing procedural control and safety. As the endoscopists' proficiency with direct intraluminal navigation and visualization improved, DV-ERAT was increasingly adopted as the first-line technique due to its superior intraluminal assessment capability. Consequently, the selection criteria shifted. The final choice depended on a combination of factors: intraoperative findings, the clarity of appendiceal visualization on ultrasound, the specific anatomical and pathological features, and the operator's judgment. UG-ERAT remained a valuable tool for complex cases or when direct entry was challenging. Throughout all procedures, a predefined protocol for aborting ERAT and converting to immediate surgery was in place, ensuring patient safety. Following the ERAT procedure, irrespective of the technical approach used, all patients entered a standardized postoperative management protocol.

### 2.6 Postoperative management

Antibiotic therapy was administered based on the intraoperative findings and pathological severity. Patients in the suppurative appendicitis group (*n* = 31) all received empirical intravenous antibiotics (third-generation cephalosporin combined with metronidazole) immediately after surgery, owing to the presence of purulent fluid or obvious suppurative changes. For patients in the simple appendicitis group (*n* = 30), antibiotic use was at the discretion of the attending physician; 23 patients (76.7%, 23/30) received antibiotics (third-generation cephalosporin and/or metronidazole), while 7 patients (23.3%, 7/30) with minimal inflammation and rapid normalization of postoperative inflammatory markers did not receive any antibiotics. The duration of antibiotic therapy was determined by clinical response (resolution of fever, normalization of white blood cell count and C-reactive protein), with a typical course of 5–7 days for uncomplicated cases and 7–10 days for suppurative cases.

Postoperatively, patients were monitored closely for abdominal pain or distension. A liquid diet was initiated 4 h after the procedure if no complications were evident. Vital signs were monitored continuously during recovery.

### 2.7 Follow-up

All enrolled patients completed the follow-up protocol, which was conducted via telephone or outpatient clinic review at predetermined intervals (1 week, 1 month, 3 months, and 6 months post-procedure). The assessments included evaluation for appendicitis recurrence, abdominal pain scores (using a validated scale), the incidence of re-consultation or readmission for related symptoms, and parental satisfaction regarding the procedure and recovery. Two patients in the suppurative appendicitis group were lost to follow-up at the 1-week time point due to incorrect contact information.

### 2.8 Outcomes

Primary Outcome Measure (Initial Technical Success):

Defined as the completion of ERAT without requiring emergency surgical intervention within 48 h post-procedure.

Secondary Outcome Measure (Comprehensive Clinical Success):

Defined as no requirement for any re-intervention (including repeat ERAT or appendectomy) due to appendicitis recurrence or related complications (e.g., abscess formation) within 180 days after the initial procedure.

Other Evaluated Parameters included: time to abdominal pain relief, incidence of procedure-related complications (e.g., bleeding, perforation), post-procedure hospital stays, and the overall recurrence rate of appendicitis.

### 2.9 Case definition and classification

The classification of appendicitis in this study followed a standardized protocol that integrated preoperative imaging findings with intraoperative endoscopic appearances observed prior to any irrigation or guidewire manipulation. The specific criteria are detailed below.

#### 2.9.1 Simple appendicitis

Diagnosis required the fulfillment of both imaging and endoscopic criteria:

Imaging Criteria: Preoperative abdominal ultrasound showed an appendiceal diameter ≥6 mm but <10 mm, with relatively preserved wall layering and only mild or no exudative changes in the peri-appendiceal fat.

Endoscopic Criteria (assessed before irrigation): Colonoscopy revealed mild congestion and edema at the appendiceal orifice, with possible spontaneous outflow of a small amount of clear or slightly turbid mucus. The mucosal vascular pattern remained largely visible, with no evidence of impacted pus or appendicoliths.

#### 2.9.2 Suppurative appendicitis

Diagnosis required the fulfillment of both imaging and endoscopic criteria:

Imaging Criteria: Preoperative ultrasound demonstrated an appendiceal diameter ≥10 mm, with wall thickening, blurred layers, intraluminal turbid fluid, or hyperechoic appendicoliths. An irregular exudative mass or localized fluid collection around the appendix was typically present.

Endoscopic Criteria (assessed before irrigation): Endoscopy showed continuous or spurting purulent discharge from the orifice. The mucosa was severely congested and erosive, with possible ulceration. Adherent pus or impacted appendicoliths were observed. Irrigation was subsequently performed for therapeutic purposes, not diagnostic confirmation.

#### 2.9.3 Definitions for outcome measures

Severe Complication: Defined as appendiceal perforation confirmed by postoperative imaging or subsequent surgery.

Recurrence: Defined as the reappearance of clinical symptoms suggestive of appendicitis (e.g., right lower quadrant pain, fever), supported by confirmatory imaging findings or surgical pathology.

### 2.10 Classification workflow

All cases were independently classified by two senior physicians (one radiologist and one endoscopist) who were blinded to the final clinical outcomes. In the event of a discrepancy, a third senior physician was consulted for arbitration. The final endoscopic classification determined the study group assignment, ensuring that categorization was based on the most direct intraluminal evidence.

### 2.11 Statistical analyses

Statistical analyses were performed using SPSS software (version 23.0, IBM Corp.). Continuous variables with a normal distribution are presented as mean ± standard deviation and were compared using the independent samples Student's *t*-test. Non-normally distributed continuous variables are expressed as median (interquartile range) and were analyzed with the Mann–Whitney *U* test. Categorical variables are reported as numbers (percentages) and were compared using the chi-square test or Fisher's exact test, as appropriate. A *post hoc* power analysis was conducted using G*Power 3.1.9.6 to assess the robustness of the findings. A two-sided *p*-value of <0.05 was considered statistically significant.

## 3 Results

### 3.1 Patient characteristics

**Figure 2:**
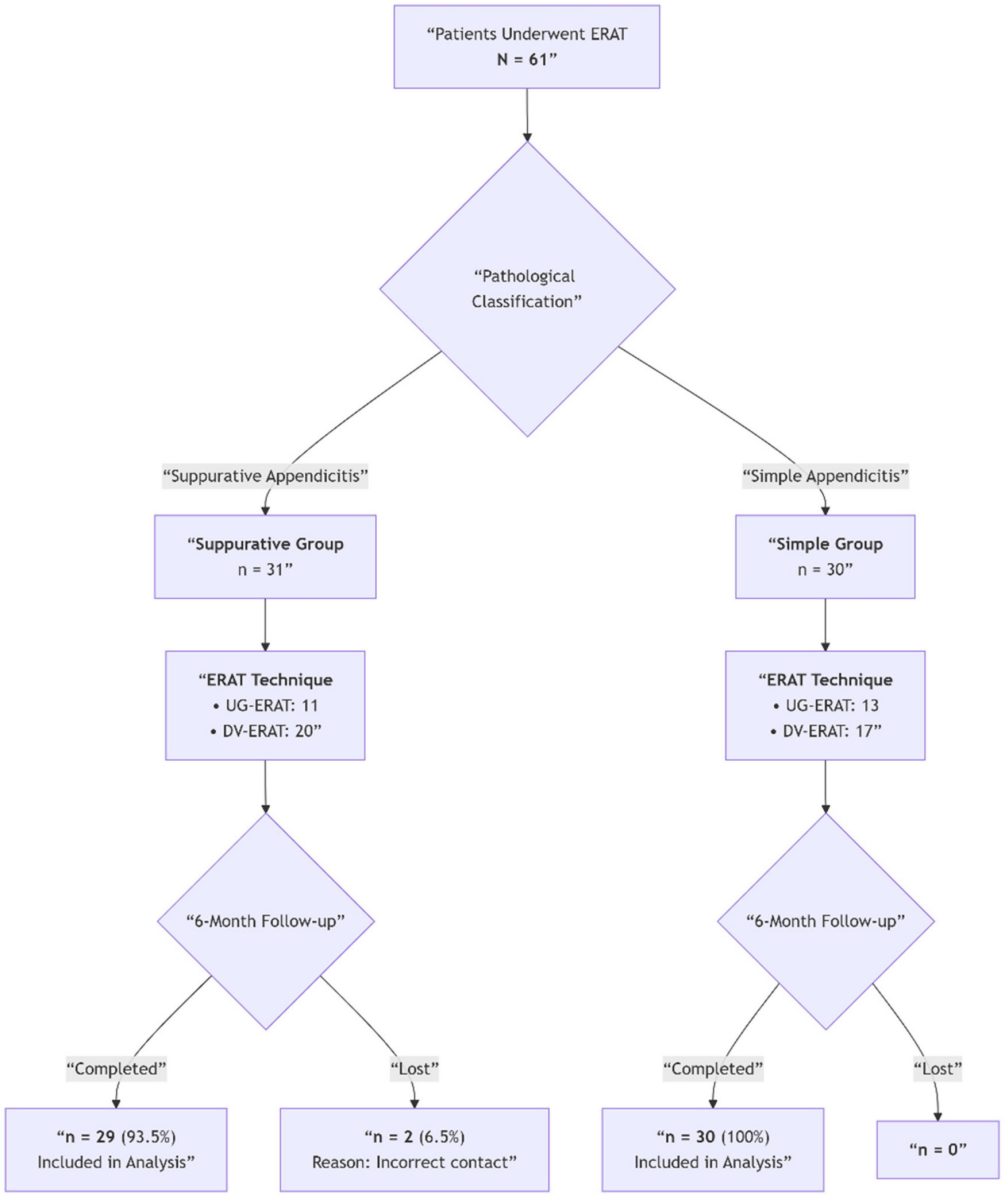
Patient flowchart from ERAT to 6-month follow-up. This diagram illustrates the progression of the 61 pediatric patients included in this retrospective cohort study. Following ERAT, patients were categorized into suppurative (*n* = 31) or simple appendicitis (*n* = 30) groups based on integrated endoscopic and imaging criteria. The flowchart details the UG-ERAT and DV-ERAT techniques within each group and tracks patient retention through the 6-month follow-up period. Two patients in the suppurative group were lost to follow-up due to incorrect contact information.

The baseline characteristics and clinical presentation of the study cohort are detailed in Table 1. The cohort comprised 61 pediatric patients who underwent ERAT, with their progression from pathological classification to follow-up completion visually summarized in Figure 2.

**Table 1:** General clinical characteristics of the Two groups of children with appendicitis.

Group	Gender (M/F)	Age (years)	ERAT Type (Ultrasound-guided/Direct Vision)	Time from Onset (d)	Abdominal Pain *n* (%)	Pain Score	Fever *n* (%)	Vomiting *n* (%)	Combined Fecalith *n* (%)	White Blood Cell Count (×10⁹/L)	CRP (mg/L)	Postoperative antibiotics, *n* (%)
Suppurative Appendicitis Group (*n* = 31)	22/9	7.66 ± 3.0	11/20	1 (1)	30 (96.7%)	3 (1)	13 (41.9%)	17 (54.8%)	13 (41.9%)	13.2 (6.37)	5.12 (14.2)	31 (100%)
Simple Appendicitis Group (*n* = 30)	18/12	8.73 ± 2.76	13/17	19 (24)	30 (100%)	7 (1)	3 (9.6%)	5 (16.1%)	5 (16.1%)	6.79 (2.85)	2.15 (2.16)	23 (76.7%)
*χ*²/t	0.812	−1.444	0.394	6.70	-	−6.768	8.036	9.634	4.68	−5.626	−3.81	-
P	0.367	0.154	0.53	0.000	1.0*	0.000	0.005	0.002	0.031	0.000	0.000	-

* Fisher's exact test.

Comparative analysis revealed that the suppurative appendicitis group exhibited significantly higher rates of fever, vomiting, and appendicoliths, along with elevated white blood cell (WBC) counts and C-reactive protein (CRP) levels, compared to the simple appendicitis group. Additionally, the preoperative pain score was significantly higher in the suppurative group than in the simple group [7 (IQR 6–8) vs. 3 (IQR 2–5), *P* < 0.001]. Symptom duration was also significantly shorter in the suppurative group (*P* < 0.05). However, there were no significant differences between the two groups in terms of age, gender, or the distribution of ERAT approaches (*P* > 0.05) (Table 1).

### 3.2 Main outcomes

Treatment outcomes by disease severity.

The mean operative time was significantly shorter in the simple appendicitis group than in the suppurative group (86.3 ± 34.4 min vs. 105.5 ± 34.1 min, *P* < 0.001).

In the simple appendicitis group, cannulation and exploration of the appendix were successful in all patients, yielding a treatment success rate of 100%. In the suppurative appendicitis group, successful cannulation and exploration were achieved in 29 of 31 cases (93.5%). Failure occurred in two patients due to excessive appendiceal curvature.

The overall treatment success rate in the suppurative group was 74.2% (23/31). Among these successful cases, seven required the placement of a plastic stent to ensure adequate drainage. Treatment failed in 6 cases: 2 due to large (>1.2 cm), hard appendicoliths that could not be retrieved endoscopically (both managed conservatively with antibiotics), and 4 due to severe intra- or post-procedural complications that necessitated conversion to surgery.

Compared with the simple appendicitis group, the suppurative group had a significantly longer median time to abdominal pain relief and extended hospital stay (all *P* < 0.05).

Two distinct ERAT techniques were employed sequentially during the study period. UG-ERAT was predominantly used in the early phase, whereas DV-ERAT became the primary approach in the later phase as operator experience accumulated.

### 3.3 Endoscopic features and postoperative outcomes of DV-ERAT

**Figure 3:**
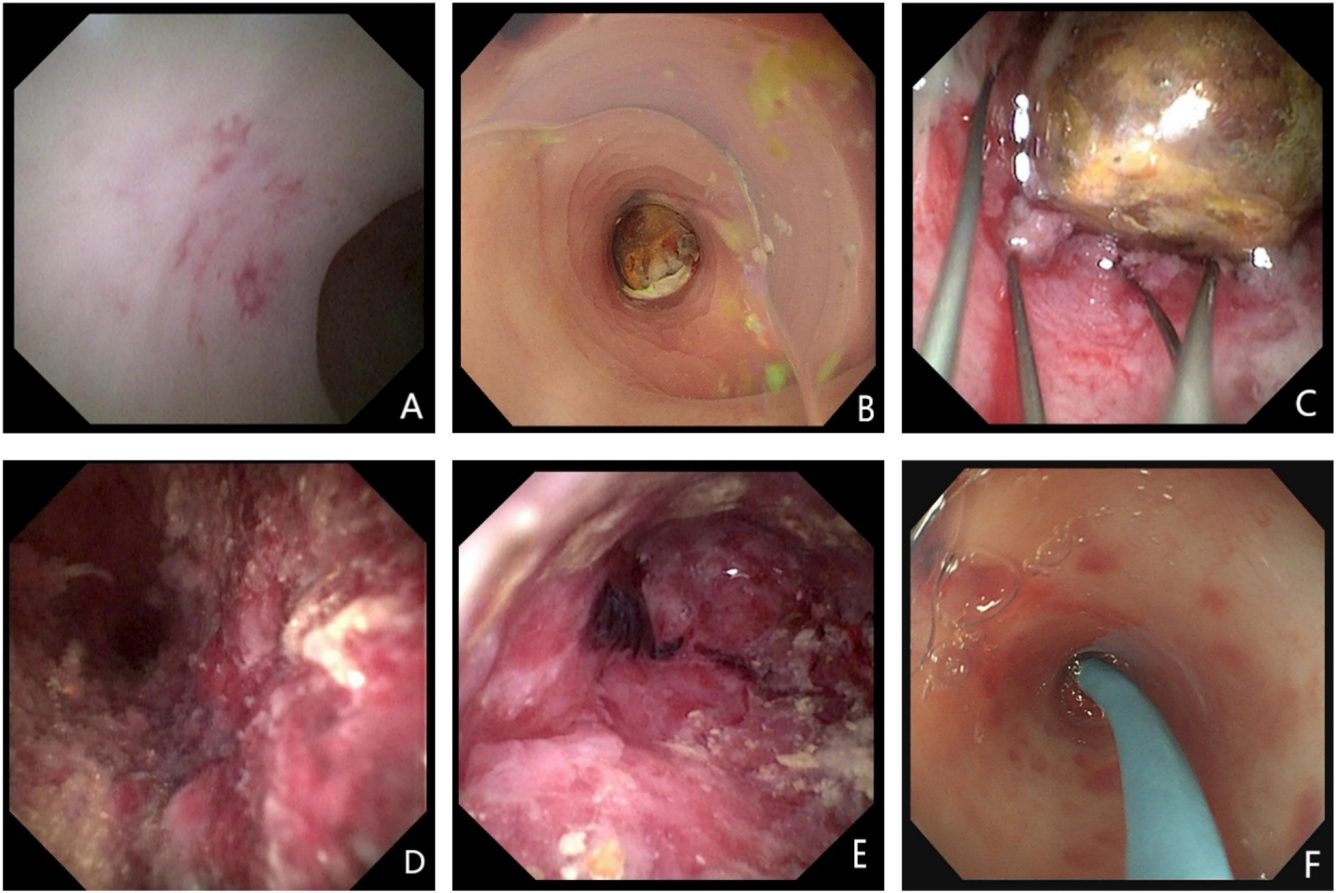
Endoscopic features of simple and suppurative appendicitis. **(A)** Simple appendicitis: patchy mucosal congestion and edema. **(B)** Suppurative appendicitis: edematous orifice with fecalith impaction and purulent discharge. **(C)** Suppurative appendicitis: impacted round fecalith within the lumen. **(D)** Suppurative appendicitis: purulent secretions and adherent fecal debris. **(E)** Suppurative appendicitis: mucosal congestion, hemorrhage, and erosion. **(F)** Post-ERAT stent placement in the appendiceal lumen.

Under direct endoscopic vision, distinct mucosal patterns were observed: in the simple appendicitis group, findings included patchy mucosal congestion and edema, occasionally accompanied by thin white exudate or fecal debris (Figure 3A); the suppurative appendicitis group exhibited more severe changes, including pronounced congestion, mucosal hemorrhage, erosion, and abundant purulent secretions or fecal debris (Figures 3B–F).

Following the procedure, the suppurative group demonstrated a significantly longer median time to abdominal pain relief, prolonged mean procedure time, and extended hospital stay compared to the simple appendicitis group. The treatment success rate was also significantly lower in the suppurative group (*P* < 0.05).

### 3.4 Comparison of complications

No severe complications occurred in the simple appendicitis group. In the suppurative appendicitis group, the most significant complication was appendiceal perforation, which occurred in 4 cases (12.9%). Among these, three perforations were identified intraoperatively and prompted immediate conversion to surgery. The remaining case presented with moderate abdominal distension during the procedure without obvious signs of perforation; however, the distension worsened 5 h after ERAT. An abdominal x-ray revealed free subdiaphragmatic air, confirming perforation, and the patient subsequently underwent surgical intervention.

### 3.5 Prognosis and follow-up outcomes

Among the 61 patients enrolled, all 30 with simple appendicitis were successfully treated with ERAT and discharged without requiring surgery. In contrast, endoscopic treatment failed in 6 patients (20.7%, 6/29) with suppurative appendicitis, necessitating conversion to appendectomy due to either cannulation failure (*n* = 2) or perforation (*n* = 4).

For patients in whom ERAT was initially successful, postoperative recovery differed markedly between groups. Patients with suppurative appendicitis experienced a significantly prolonged recovery course, with a median time to pain relief of 6 days (IQR 1) vs. 2 days (IQR 1) and a median hospital stay of 10 days (IQR 4) vs. 7 days (IQR 1.25) in the simple appendicitis group (both *P* < 0.001; Table 2). Parental satisfaction scores during follow-up were consistently lower in the simple appendicitis group, while abdominal pain scores were higher in the suppurative group, although these differences did not reach statistical significance at most time points (all *P* > 0.05; Table 2).

**Table 2:** Comparative postoperative outcomes and follow-up between simple and suppurative appendicitis groups treated with ERAT.

Outcome Measure	Simple Appendicitis Group (*n* = 30)	Suppurative Appendicitis Group (*n* = 31)	*P* Value
Procedural Outcomes			
Operative Time (minutes), mean ± SD	86.3 ± 34.4	105.5 ± 34.1	<0.001
Primary ERAT Success, *n* (%)	30 (100.0)	23 (74.2)	<0.001
Conversion to Appendectomy, *n* (%)	0 (0.0)	6 (20.7)	0.001
Due to Cannulation Failure	0	2	—
Due to Perforation	0	4	—
Early Recovery Outcomes			
Median Time to Pain Relief, days (IQR)	2 (1)	6 (1)	<0.001
Median Hospital Stay, days (IQR)	7 (1.25)	10 (4)	<0.001
Follow-up Outcomes			
Abdominal Pain Score, median (IQR)			
1 Week	0 (0, 0.5)	1 (1, 2)	0.389
1 Month	0 (0, 0)	1 (1, 2)	0.285
3 Months	0 (0, 0)	1 (1, 2)	0.085
6 Months	0 (0, 0)	1 (1, 2)	0.085
Parental Satisfaction Score, median (IQR)			
1 Week	2 (2, 2)	1 (1, 2)	0.288
1 Month	2 (2, 2)	1 (1, 2)	0.35
3 Months	2 (2, 2)	1 (1, 2)	0.241
6 Months	2 (2, 2)	1 (1, 2)	0.241
Recurrence at 6 Months, *n*/*N* (%)	3/30 (10.0)	5/23 (21.7)	0.117

Regarding the primary long-term outcome, recurrence rates at 6 months were 21.7% (5/23) in the suppurative group and 10.0% (3/30) in the simple group, a difference that was not statistically significant (*P* = 0.117; Table 2). Recurrence rates at other follow-up time points also showed no significant intergroup differences (all *P* > 0.05; Table 2). A *post hoc* power analysis for these recurrence comparisons indicated low to moderate statistical power (ranging from 10.4% to 55.2% across time points). Detailed comparative outcomes are presented in Table 2.

## 4 Discussion

This retrospective study yielded three principal findings: (1) ERAT is safe and highly effective for pediatric simple appendicitis, achieving a 100% success rate without major complications; (2) for suppurative appendicitis, ERAT is associated with a substantially lower success rate (79.3% 23/29) and a clinically important perforation risk (13.8% 4/29), and a significantly longer mean operative time (105.5 ± 34.1 min vs. 86.3 ± 34.4 min, *P* < 0.001); and (3) prolonged symptom duration (>48 h) and the presence of an appendicolith were frequently observed in cases of technical failure or complication.

The favorable outcomes observed in the simple appendicitis cohort are consistent with and extend prior findings (12–14, 16–19). Although ERAT has been established as effective in large adult populations-including a Chinese multi-center study of 3,196 patients reporting a >90% success rate (20), pediatric-specific evidence remains limited. This study provides dedicated pediatric evidence for ERAT outcomes stratified by appendicitis severity. Our findings-rapid symptom resolution, brief hospitalization, and no recurrences requiring surgery-mirror the efficacy profile established in adults, supporting the feasibility and effectiveness of ERAT in children. This cross-age consistency reinforces the generalizability of the technique and offers clinically actionable data to inform decision-making and guide future pediatric trials. These results further substantiate ERAT as a viable, organ-preserving first-line strategy for uncomplicated pediatric appendicitis, balancing therapeutic efficacy with minimal invasiveness (14, 15).

The significantly longer mean operative time in the suppurative group (105.5 ± 34.1 min vs. 86.3 ± 34.4 min, *P* < 0.001) reflects the increased technical difficulty posed by severe inflammation, luminal debris, and distorted anatomy. The significantly poorer performance of ERAT in suppurative appendicitis can be attributed to its distinct pathophysiology. Suppurative inflammation involves neutrophilic infiltration, mucosal ulceration, and purulent exudate, which collectively weaken the appendiceal wall (21, 22). This altered tissue integrity increases vulnerability to perforation during endoscopic manipulation, irrigation, or stent placement (23). Furthermore, the inflammatory edema and luminal debris more frequently obscure anatomy and impede complete clearance, explaining the higher rates of technical failure and incomplete treatment leading to recurrence. These pathophysiological distinctions not only explain the observed outcome disparity but also underscore the imperative for refined patient selection to optimize the risk-benefit profile of ERAT.

This 6-month recurrence rate of 21.7% (5/23) is based on a follow-up rate of 93.5% (29/31) in the suppurative group, lending robustness to this observation despite the lack of statistical significance. The identified risk factors-namely, symptom duration >48 h and the presence of an appendicolith-provide actionable insights for clinical decision-making and support our rationale for selecting a 48-hour primary endpoint. Prolonged symptoms often correlate with more advanced inflammation and tissue necrosis, increasing procedural difficulty and risk (22). Appendicoliths, particularly those larger than 1.2 cm or impacted in a tortuous appendix, pose a direct mechanical challenge. They can cause persistent obstruction, are difficult to extract endoscopically, and their removal attempts significantly elevate perforation risk (23). These factors validate the critical 48-hour window for assessing whether ERAT can avert emergency surgery by interrupting the acute inflammatory cascade before these high-risk features lead to irreversible progression. Consequently, these factors must be integral to preoperative counseling and shared decision-making. They serve as critical indicators that may tilt the risk-benefit calculus towards primary appendectomy for certain patients, while also identifying cases where ERAT, if attempted, requires utmost technical caution and preparedness for conversion. The significantly longer recovery time and hospital stay observed in the suppurative group, coupled with the trend toward lower parental satisfaction scores, further inform this risk-benefit calculus. These factors emphasize that even when technically successful, ERAT for suppurative appendicitis entails a more burdensome recovery process, which should be clearly communicated during shared decision-making.

Our study confirms a distinct efficacy gap between simple and suppurative appendicitis, a finding consistent with prior literature (24). The observed variability in success and complication rates across studies likely reflects heterogeneous patient populations, evolving technical protocols, and differences in operator experience. Notably, our own technical evolution from a primary reliance on UG-ERAT to the predominant use of DV-ERAT offers a critical lens through which to interpret outcomes in complex cases. In the early phase, UG-ERAT was instrumental for navigating difficult anatomy and confirming wire placement in markedly distended or obstructed appendices, which were more common in the suppurative group. As proficiency with DV-ERAT grew, its superior ability to provide direct intraluminal assessment of mucosal integrity, pinpoint the source of obstruction (e.g., small or impacted appendicoliths), and visually confirm complete clearance became invaluable. This technical shift likely contributed to improved decision-making in the suppurative group over time; for instance, direct visualization of severe mucosal necrosis or a tenaciously impacted stone could prompt an earlier and more deliberate decision to abort the procedure and convert to surgery, thereby potentially averting a blind, high-risk manipulation that might lead to perforation. Consequently, our reported perforation rate, while significant, may reflect a learning curve where enhanced visualization helped identify rather than cause procedural limits in high-risk anatomy. This underscores that technical choice is not static but should be dynamically adapted to case complexity, and it highlights the urgent need for standardized benchmarks to define operator proficiency and procedural endpoints, especially in managing suppurative appendicitis.

The clinically notable recurrence rate of 21.7% (5/23) at 6 months in the successfully treated suppurative appendicitis cohort warrants careful consideration. This rate, though not statistically different from the simple appendicitis group in our study, is numerically substantial and may reflect the distinct pathophysiology of suppurative inflammation. Potential explanations include: (1) incomplete eradication of the nidus of infection due to severe mucosal edema and purulent debris, which may hinder complete endoscopic clearance and allow residual bacteria to persist; (2) impaired mucosal healing in a necrotic or ulcerated lumen, potentially leading to persistent low-grade inflammation or luminal stenosis; and (3) the possibility of undetected micro-perforations or persistent obstruction in a subset of cases. From a clinical management perspective, this underscores the necessity of enhanced post-procedural surveillance for patients with suppurative appendicitis treated by ERAT. Close follow-up within the first 3–6 months, including symptom assessment and possibly interval imaging, may be prudent to identify early recurrence. Furthermore, this finding reinforces the argument for selective stenting in suppurative cases, as prolonged drainage via a stent may facilitate more complete resolution of inflammation and reduce recurrence risk, a strategy that was employed in seven of our successful cases. Future studies should aim to identify predictors of recurrence to optimize patient selection and post-ERAT management protocols.

Recent studies report 20%–30% recurrence rates for antibiotic-treated uncomplicated appendicitis (25, 26), which closely mirror our 6-month recurrence rate in the suppurative group (21.7%). However, ERAT offers a theoretical advantage over antibiotics alone by directly addressing luminal obstruction-removing appendicoliths and draining purulent debris to achieve complete source control. Antibiotics may be insufficient when persistent obstruction creates an ongoing infection nidus, leaving patients at risk for perforation (25). Whether these theoretical benefits translate into superior clinical outcomes remains unclear. ERAT requires anesthesia and carries procedural risks, while antibiotic therapy avoids invasiveness but leaves the appendix *in situ* with uncertain long-term fate. Future randomized trials comparing ERAT vs. antibiotic therapy stratified by severity and appendicolith presence are needed to define optimal treatment algorithms.

As an emerging technique, ERAT shows considerable promise but also faces significant challenges. It demonstrates high efficacy in pediatric cases of simple appendicitis; however, its success rate is lower and the risk of perforation higher in suppurative cases, underscoring the need for careful patient selection based on symptom duration and appendicolith characteristics. The development of a dedicated preoperative assessment system potentially incorporating clinical symptoms, laboratory markers, imaging features, and procedural factors into a prognostic scoring tool is essential for improving risk stratification and outcome prediction.

Regarding procedural time and anesthesia, we acknowledge that ERAT requires general anesthesia in children and involves a longer operative time than laparoscopy. Our data confirm that ERAT is particularly time-consuming in suppurative cases, with a mean operative time of 105.5 min. However, the definition of “minimally invasive” in pediatric surgery extends beyond incision size and procedural duration to include organ preservation and long-term quality of life. The appendix is now recognized as an immune organ with potential roles in gut flora homeostasis. Thus, the trade-off for longer procedural time and general anesthesia is the benefit of appendiceal preservation and a completely scarless outcome. For carefully selected patients with simple appendicitis, where the procedure is relatively swift (mean 86.3 min) and highly successful, this trade-off may be favorable. Future efforts should focus on refining endoscopic instruments and techniques to shorten procedural duration in children.

The findings of this study should be interpreted within the context of several important limitations. First, its retrospective, single-center design and the modest sample size particularly within the suppurative appendicitis subgroup (*n* = 31) limit the statistical power for several key comparisons. *post hoc* analysis revealed low to moderate power (10.4%–55.2%) for the comparison of recurrence rates between groups. Consequently, while the observed difference in recurrence (21.7% vs. 10% at 6 months; *P* = 0.117) did not reach statistical significance, this may reflect a type II error. This numerical trend should thus be considered an exploratory, hypothesis-generating finding that requires validation in larger cohorts. Similarly, analyses of complication subtypes or risk factors were underpowered and are preliminary. Second, and most critically, inherent selection bias must be acknowledged. Our cohort comprised children pre-selected as suitable ERAT candidates through multidisciplinary assessment, excluding those with severe presentations (e.g., diffuse peritonitis, septic shock), imaging suggesting perforation or gangrene, or parental preference for immediate surgery. Consequently, our study population represents a highly selected cohort with predominantly mild disease. This selection bias is particularly relevant to our primary conclusion: the favorable outcomes reported here reflect the efficacy and safety of ERAT in an optimally selected population. They likely underestimate the true complication rates, specifically the perforation risk, if ERAT were applied to an unselected spectrum of suppurative appendicitis. Therefore, our findings strongly support reserving ERAT for simple appendicitis, and the significant risks observed in the suppurative subgroup, even in this selected cohort, reinforce our recommendation against its use in complicated cases. Third, the technical protocol evolved during the study period, shifting from initial reliance on UG-ERAT to the preferential use of DV-ERAT. While this mirrors the natural progression of clinical expertise, it introduces heterogeneity in procedural data over time, including potential variations in operative time, a factor this study was not designed to analyze. Fourth, the prolonged hospital stay (median 7–10 days) raises concerns regarding ERAT's cost-effectiveness. This reflects conservative discharge practices and the reality that many patients traveled from distant areas, making early follow-up impractical. Future protocols should focus on evidence-based early discharge criteria to shorten hospitalization and improve cost-effectiveness. Finally, the low recurrence event rate precluded reliable multivariate analyses. Thus, all identified associations between potential risk factors and outcomes should be regarded as exploratory and require confirmation in larger studies.

To address these limitations and advance the field, future research should focus on prospective, multi-center studies with larger cohorts. Such studies are needed to prospectively define precise eligibility criteria for ERAT and, ultimately, to conduct randomized controlled trials comparing ERAT with early laparoscopic appendectomy. This will be essential for a more comprehensive and generalizable assessment of its real-world efficacy, safety, and risk profile.

For patients with suppurative appendicitis, the decision to use ERAT warrants careful consideration. While ERAT can help reduce intraluminal pressure and alleviate symptoms, potentially creating more favorable conditions for subsequent surgery, its application in this setting requires prudent judgment. In summary, ERAT is a safe and effective organ-preserving option for carefully selected pediatric patients with simple appendicitis. However, given the significantly lower success rate and clinically meaningful perforation risk observed in the suppurative group, we do not recommend ERAT for cases of suppurative appendicitis accompanied by imaging findings suggestive of a thin-walled appendix or a large fecalith. In such instances, laparoscopic appendectomy remains the standard of care. Therefore, appropriate patient selection is critical; ERAT should be reserved for cases of simple appendicitis and select instances of non-necrotizing fecalith impaction.

## Data Availability

The raw data supporting the conclusions of this article will be made available by the authors, without undue reservation.
